# Advances in the Genetically Engineered KillerRed for Photodynamic Therapy Applications

**DOI:** 10.3390/ijms221810130

**Published:** 2021-09-20

**Authors:** Jiexi Liu, Fei Wang, Yang Qin, Xiaolan Feng

**Affiliations:** Key Laboratory of Medicinal Resources and Natural Pharmaceutical Chemistry, Ministry of Education, National Engineering Laboratory for Resource Developing of Endangered Chinese Crude Drugs in Northwest of China, College of Life Sciences, Shaanxi Normal University, Xi’an 710119, China; liujiexi2021@163.com (J.L.); feiwang0705@163.com (F.W.); qy971023@163.com (Y.Q.)

**Keywords:** photodynamic therapy, genetically encoded photosensitizers, KillerRed, delivery strategies, nanotechnology

## Abstract

Photodynamic therapy (PDT) is a clinical treatment for cancer or non-neoplastic diseases, and the photosensitizers (PSs) are crucial for PDT efficiency. The commonly used chemical PSs, generally produce ROS through the type II reaction that highly relies on the local oxygen concentration. However, the hypoxic tumor microenvironment and unavoidable dark toxicity of PSs greatly restrain the wide application of PDT. The genetically encoded PSs, unlike chemical PSs, can be modified using genetic engineering techniques and targeted to unique cellular compartments, even within a single cell. KillerRed, as a dimeric red fluorescent protein, can be activated by visible light or upconversion luminescence to execute the Type I reaction of PDT, which does not need too much oxygen and surely attract the researchers’ focus. In particular, nanotechnology provides new opportunities for various modifications of KillerRed and versatile delivery strategies. This review more comprehensively outlines the applications of KillerRed, highlighting the fascinating features of KillerRed genes and proteins in the photodynamic systems. Furthermore, the advantages and defects of KillerRed are also discussed, either alone or in combination with other therapies. These overviews may facilitate understanding KillerRed progress in PDT and suggest some emerging potentials to circumvent challenges to improve the efficiency and accuracy of PDT.

## 1. Introduction

**Figure 1:**
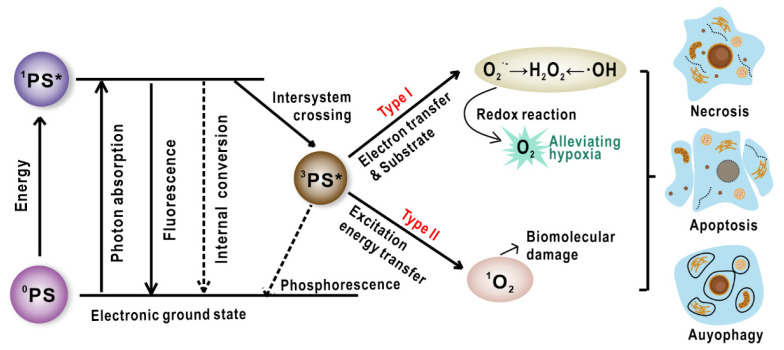
The different mechanisms of photosensitization processes and the resultant variable cell death modes (software: CorelDraw 2020, 22.0.0.412). *: Electronic excited state.

Photodynamic therapy (PDT), as a non-invasive modality with spatiotemporal selectivity, has been used to treat a variety of cancers as well as non-oncological indications like infections and dermatoses [1,2,3,4,5]. Light, photosensitizers (PSs), and oxygen are the three key elements for PDT, which lack toxicity in individual conditions but produce toxicity when they work together. After exposing PSs to a particular wavelength of light, photochemical reactions produce reactive oxygen species (ROS) that can cause irreversible oxidization on essential cellular components and result in neoplastic cell death through apoptosis, necrosis, autophagy, degeneration, and inflammatory response in the treated area [6,7]. In the photoactivation process, the excited triplet state of PSs either interact with biomolecules in the surrounding environment by transferring electrons and result in free radical generation such as superoxide ion and hydroxyl radical that destruct biomolecules (called type I reaction), or directly transfer energy from the triplet PSs towards oxygen, resulting in the production of singlet oxygen (^1^O_2_) (type II reaction) (Figure 1) [6]. The contributions of type I and type II mechanisms are affected by a variety of factors including pH value, tissue dielectric constant, oxygen concentration, and the properties of PSs. Since PDT can rapidly consume lots of tissue oxygen and also shut down the blood vessels that deliver oxygen, the treatment may induce more serious hypoxia in tumor environment [8]. Although the details of how oxygen involves in type I reaction are still unclear, many studies suggest that type I PDT works well even under scarce oxygen conditions. Thus, type I may provide new solutions to overcome the hypoxia dilemma in neoplasms treatment [9].

PDT is clinically appealing owing to its minimal invasiveness, locoregional therapies, limited side effects, repeatable stimulation, and negligible resistance. Undoubtedly, PSs are crucial for the highly efficient PDT and many efforts have been devoted to developing photosensitive compounds. PSs have evolved from the first-generation PSs such as haematoporphyrin derivative (HpD) and Photofrin to the second-generation PSs such as chlorines, phthalocyanines, and some dyes, or even the third-generation PSs centered on the development of substances that have a stronger affinity for tumor tissue [10]. Some of the previous PSs have been approved for clinical application, but they are still demonstrated several shortcomings. For instance, most porphyrins have good photophysical properties, but they tend to aggregate in biological media because of the enhanced π-stacking of macrocycles. Based on this, nanophotosensitizers including various types of nanoparticles such as organic liposomal porphysome, inorganic titanium dioxide nanoparticles, and other nanohybrids have been developed in recent years. Nevertheless, the nanosized PSs with complicated fabrications are still a long way from clinical use. Moreover, most of the synthesized PSs are thought to act through type II reactions to cause oxidative cellular damage, while the type I PSs may be able to produce more effective PDT effects within an anoxic tissue environment.

**Figure 2:**
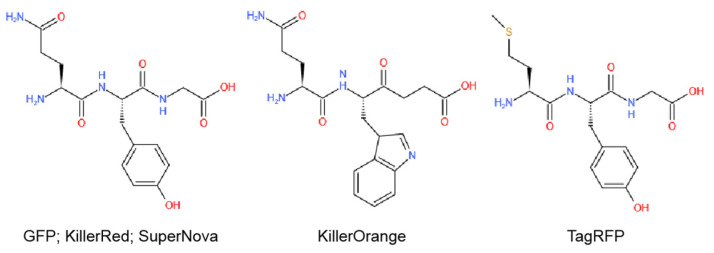
The main chromophores in different fluorescent proteins (software: Kingdraw).

Excitedly, some fluorescent proteins including GFP, KillerRed, KillerOrange, TagRFP, SuperNova, miniSOG, and their ramifications have been proved to possess photosensitive properties at different levels (Figure 2, Table 1). More importantly, by using genetic modification techniques, they can be genetically encoded to achieve precise cell/tissue distribution and perform PDT reactions [11]. Unlike conventional fluorescent proteins like GFP and TagRFP with inefficient photosensitization properties, KillerRed is the first phototoxic fluorescent protein designed by Bulina’s team in 2006 [12]. KillerRed is derived from the non-fluorescent chromoprotein in anm2CP via the substitutions Thr145Asp and Cys161Gly, which creates a water-filled channel connected with chromophore through the center of β-barrel. This structure is unique to KillerRed and may be the principle for its superior phototoxicity. It has been reported that KillerRed can target a particular organelle or compartment by fusing a localizing sequence (e.g., leader peptides or antibodies). Moreover, CALI (chromophore-assisted light inactivation) and photoablation studies have also made extensive use of KillerRed to investigate the subcellular structure and function by optogenetics. To further develop the functions and applications of KillerRed, this review will focus on the structure, reaction mechanism, and physiological function of KillerRed as both endogenous and exogenous PS for cancer therapeutics and imaging. The phototoxicity and future perspectives of KillerRed, as well as the combination with chemotherapy/gene therapy/nanoparticles, has also been discussed.

## 2. Basic Features of KillerRed

### 2.1. Structure and Property of KillerRed

**Figure 3:**
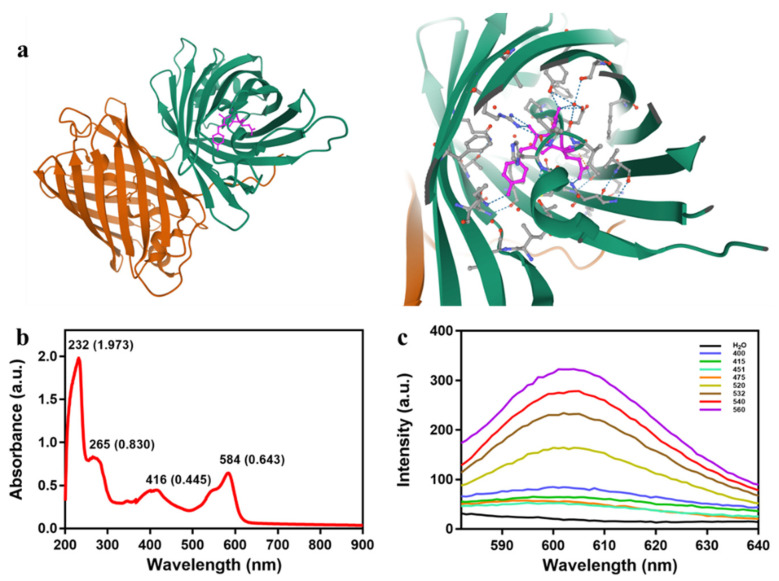
(**a**) Stereoview of the cavities occurring in the dimeric KillerRed; the chromophore is colored in pink, and the hydroxy benzylidene group is shown in orange. Download from the NCBI website (1 November 2020): https://www.ncbi.nlm.nih.gov/Structure/icn3d/full.html?&mmdbid=81360&bu=1&showanno=1&source=full-feature. (**b**) Absorption spectrum of KillerRed. (**c**) Fluorescence spectra of KillerRed excited by visible light at different wavelengths.

KillerRed is a dimer consisting of GFP-like β-barrel with a typical chromophore (Gln65-Tyr66-Gly67) through the β-barrel axis, wherein a unique water-filled channel forms there (Figure 3a). The chromophore can absorb 540~580 nm wavelength green light and emit a longer 610 nm red light (Figure 3b,c), which facilitates the production of ROS trough exchanging oxygen and ions with the surrounding environment to induced phototoxicity [13,15,30]. It is shown that the maximum fluorescence excitation/emission of KillerRed is 585/610 nm, and the fluorescence quantum yield of KillerRed is 0.25, which is 62.5 times higher than that of GFP. Furthermore, the phototoxicity of KillerRed has exceeded other fluorescent proteins by at least 1000-fold [15]. At present, the type I photoreaction induced by KillerRed is widely approved, suggesting that KillerRed may be adaptable to the hypoxic microenvironment in tumor tissues.

### 2.2. Illumination Factors of KillerRed

Lasers emitting precise amounts of light are widely used in phototherapy. As the wavelength stretches, the time required to achieve the same effect increases. Since PDT consumes oxygen, it is critical to use an acceptable irradiance. Otherwise, a high irradiance will consume the oxygen molecules too quickly, resulting in a decrease in efficiency [7]. Furthermore, the photodamage to cells is influenced by the various laser parameters used in the experiments, such as repetition rate, pulse frequency and light intensity. Experiments revealed that a pulsed laser (584 nm, 10 Hz, 18 ns) induced major histopathological changes and slowed the growth of a CT26 transplanted tumor, while a continuous laser (593 nm) had little effect [31]. Meanwhile, KillerRed can also present different cellular responses after treatment by disparate light intensities. For example, KillerRed expressed on the surface of lysosomes triggered cell necrosis via a higher light intensity (700 mW/cm^2^, 5 min), but mediated cell apoptosis at a lower light intensity (75 mW/cm^2^, 20 min) [32]. Based on the above studies, it could be postulated that low light intensity is feasible in KillerRed-mediated PDT for cell death.

## 3. Applications of KillerRed as an Endogenous Photosensitizer

With the development of modern biomedicine, researchers have been devoted to investigate precision PDT strategies. It knows that ROS has a short life span, which makes it only react with biomolecules within a micron range. Therefore, the therapeutic effeciency and mechanisms are highly dependent on the intracellular localization of PSs. In this regard, the location of KillerRed can be readily modified by the genetic engineering to enhance oxidative damage of specified organelles. 

### 3.1. Distinct Targeting Strategies of KillerRed In Vitro

PDT damages cancer cells not only directly by apoptotic and non-apoptotic (necrosis, autophagy) pathways, but also indirectly by disrupting tumor vasculature that supports cancer cells with nutrients and oxygen. Therein, the type of PS and localization are crucial for the different damage pathways induced by PDT. Previous studies have showed that PSs located in mitochondria are more likely to cause apoptosis and PSs distributed in the plasma membrane and lysosomes cause necrosis. Unlike chemical PSs, genetically encoded PSs can be modified and targeted to a specific cellular compartment or cell type using genetic engineering techniques [32]. Enhancing the spatiotemporal interaction between PSs and their designated target will significantly improve PDT therapeutic efficacy. In this section, we will introduce different targeting strategies and related applications of KillerRed (Table 2).

#### 3.1.1. Membrane-Targeted KillerRed

The plasma membrane plays a crucial role in maintaining cellular homeostasis, cell integrity, and nutrient transport [52]. Cellular life will undoubtedly come to an end if the plasma membrane’s integrity is compromised [52]. ROS can lead to unsaturated lipid peroxidation then cause lipid membrane conformational changes and eventually programmed necrosis. As a result, membrane-targeted PDT will be a potent strategy for disrupting cellular integrity.

**Figure 4:**
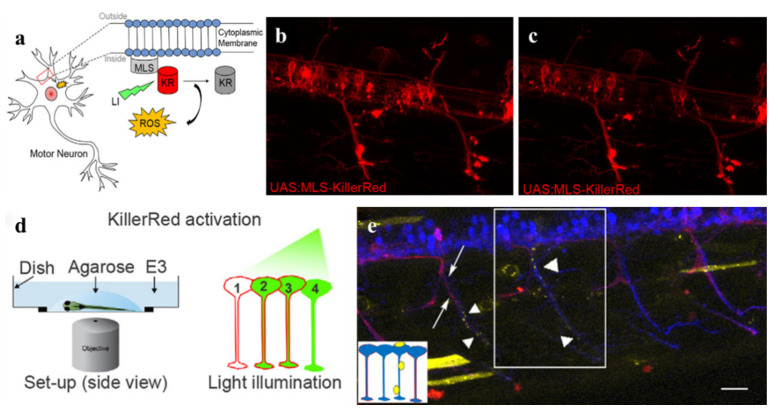
(**a**) The membrane localization signal (MLS) directs KillerRed (KR) to the intracellular cell membrane of MNs (mnx1 promoter). Following green light illumination, KR produces ROS and photo-bleaching occurs. (**b**) A transgenic zebrafish can express KR in individual neurons. (**c**) Photo-bleaching occurs after 60 min of illumination for KR. (**d**) A schematic representation of mem-KR activation in zebrafish. (**e**) Time-lapse imaging after KR activation showed A5 (a physiological marker of apoptosis) accumulation. Scale bars 25 µm. Reproduced with permission from Ref. [33]. Copyright 2018 Elsevier.

Oxidative stress is linked to a variety of diseases, including cardiovascular, cancer, and neurodegenerative diseases. In previous studies, the standard approach caused oxidative stress has been applicating ROS-generating reagents globally which may bring systemic side effects and safety issues. Thus, specific neuronal ablation is necessary to kill cells as quickly as possible without collateral damage to adjacent cells and tissues [38]. To achieve this, combining photosensitizing proteins with PDT opens up a novel therapeutic modality. Optogenetics is an emerging field to accurately manipulate cells activity by using molecular genetics to express light-sensitive proteins [33]. It also achieves advanced temporal and spatial regulation of oxidative stress production. Studies suggested that ROS can regulate the degeneration and ablation of motor neurons and sensory neurons in zebrafish and *Caenorhabditis elegans* by establishing models in vivo and expressing real-time visualized membrane-targeted KillerRed (mem-KR) selectively (Figure 4). The results hint that oxidative stress has close relation with neurodegeneration and KillerRed-mediated optogenetics is useful for behavioral analysis and genetic research [33,38,39]. Optogenetics also provides new opportunities for exploring the mechanism of biological development, regeneration, and repair. Photo-activated mem-KR can induce changes in heart rate and contractility of zebrafish. Moreover, it can produce oxidative stress in *X.laevis* tadpoles and help to investigate the conserved mechanisms of cardiac repair during natural heart morphology reconstruction [34,36]. It can affect cell viability and function of the zebrafish embryo as well as induce apoptosis in specific organs and tissues of *Xenopuslaevis* for studies of ROS during embryogenesis [35,37].

In cancer therapy, the plasma membrane remains an essential target for novel drugs, as the majority of exogenous substances are readily stuck in enzyme degradation following cellular internalization by acidic endo/lysosomal compartments [53,54]. However, encoded membrane-targeted KillerRed can overcome different biological obstacles and achieve efficient PDT without endocytosis. Therefore, we can expect in further studies that mem-KR will play an important role in targeting tumor ablation.

#### 3.1.2. Mitochondria-Targeted KillerRed

As the essential organelle in cell energy metabolism, ROS formation and the control of programmed cell death (PCD) [55,56], mitochondria are very vulnerable to ROS because their contents are likely to cause oxidative damage in the matrix [44,57]. Multiple PSs such as porphyrin derivatives, chlorin e6 (Ce6), curcumin, Zn (II) Phthalocyanine (ZnPc), cyanine dyes, etc. have been designed to accumulate in mitochondria by combining with targeting agents [58]. Compared to the above synthetic compounds, the fluorescent protein KillerRed can be easily modified to target mitochondria by inserting an MTS (mitochondria localization signal) sequence into the genome of KillerRed.

Neuronal mitochondria play important roles in neuronal physiology, but the relationship between neuronal and mitochondrial dysfunction is still unclear. One research has indicated that mitochondria-targeted KillerRed (mt-KR) and mem-KR function through two different pathways: Photoactivated mt-KR resulted in organelle fragmentation without killing the cells, while mem-KR caused cell death via lipid peroxidation [38]. Several potential reasons may contribute to this phenomenon of mt-KR. First, the antioxidant system in mitochondria can counteract oxidative stress, and induce a low rate of effective ROS diffusion and rapid quenching of ROS. Second, the defective mitochondria show a lower motility and fusion, and avoid the propagation of oxidation in the neuron by “quarantining” themselves [42]. Third, the function of proteasomes degrades the activated caspase-3 to limit the spread of caspase-3 activity and cell death [41]. Additionally, the mitochondrial damage induced by mt-KR-mediated PDT further results in impaired muscle function and interference to feeding and development of *C. elegans* larvae [40].

Except in the neurons and nematodes, mitochondria are also closely related to cancer cells survival and death. Mt-KR has shown remarkable effects against cancer cells through different pathways such as caspase-dependent or -independent cell apoptosis, and cell autophagy. The mechanisms can be summed up as the following points: First, oxidative stress caused by photo-stimulated mt-KR can increase the permeability of mitochondrial membrane and release cytochrome C to activate the caspases pathway, which finally leads to cell apoptosis [40,41,43]. Second, mt-KR can induce caspase-independent cell death after illumination via mitochondrial membrane depolarization, the generation of ROS increase and mitochondrial rupture/dysfunction [40]. Third, mt-KR-mediated phototoxicity can initiate PARK2/PARKIN-dependent mitochondrial autophagy, leading to autophagic cell death [44]. Moreover, studies have shown that linear mitochondria are more resistant to mitophagy than broken mitochondria by unclear mechanisms.

Overall, mt-KR-mediated PDT is a promising therapeutic strategy for various diseases because it can overcome additional barriers such as the nuclear membrane and avoid unexpected leakage. It is still beneficial to explore the mechanism of different cell death. However, it has been reported that basal expression of mt-KR in the muscle cells of worms can cause mitochondrial stress and induce delayed growth and development, even in the absence of irradiation with light, which is a limitation for the mt-KR application [40]. Therefore, ensuring the safety of mt-KR expression and improving treatment efficiency are still major challenges.

#### 3.1.3. Nucleus-Targeted KillerRed

The cell nucleus is the main target for many therapies such as chemotherapy, gene therapy, PDT, and PTT. The endogenous KillerRed can be designed to target the nucleus by fusing various nuclear localization sequences. After light stimulation, ROS generated from nucleus-targeted KillerRed (nuc-KR) can directly cause DNA damage with precise temporal and spatial control when compared to chemical PSs.

Studies have indicated that the photoactivated nuc-KR can induce premature senescence. Compared to oncogene-induced senescence (OIS) and senescence induced by DNA-damaging agents, nuc-KR with light activation can avoid interference to the cell culture and side effects of small-molecule drugs [51]. By expressing KillerRed in series with histone 2B (H2B) within the HeLa cells, the fusion protein H2B-KR can induce DNA damage and further lead to cell senescence after illumination. Telomeres are also strongly associated with aging, but the studies of realizing precise oxidative damage to telomeres remain inadequate [50]. Telomere-targeted KillerRed (tel-KR) provides a new targeting strategy for exploring the relationship between telomeric oxidative damage and aging. Li Lan et al. have designed tel-KR by fusing KillerRed with TRF1 (telomeric repeat-binding factor 1) to induce specific telomeric oxidative damage and revealed the mechanism of telomere protection: TRF1 is phosphorylated and preserved in a functional shelterin complex at telomeres by the Nek-7 (one of the never-kinase family Mitotic gene A) [35,50,51].

In addition to cellular senescence, oxidative stress caused by activated nuc-KR is also an efficient approach for tumor elimination. Previous studies have demonstrated that KillerRed fusing with histone 2A (H2A) or nuclear lamina protein B1 can trigger cell cycle arrest, increase the rupture of the DNA strand, and eventually kill the tumor cell [45,46,59]. Moreover, nuc-KR fusing with a tet-repressor (tetR) or transcription-activator (TA) in U2OS cells can produce ROS and cause heterochromatin or euchromatin damage after illumination. Since the ROS-induced DNA damage can be repaired by the base excision repair (BER) pathway, nuc-KR is useful to investigate how the BER protein is recruited to DNA damage sites in cells [47,48].

The nucleus is an effective target of PDT because the DNA double-strand damage is lethal to cells [49]. Notably, compared to other exogenous photosensitizers, endogenous nuc-KR can achieve specific DNA damage. However, how the KillerRed precisely induced DNA damage and the regulated mechanism are yet to be fully elucidated.

### 3.2. Diverse Delivery Strategies of KillerRed Gene In Vivo

**Figure 5:**
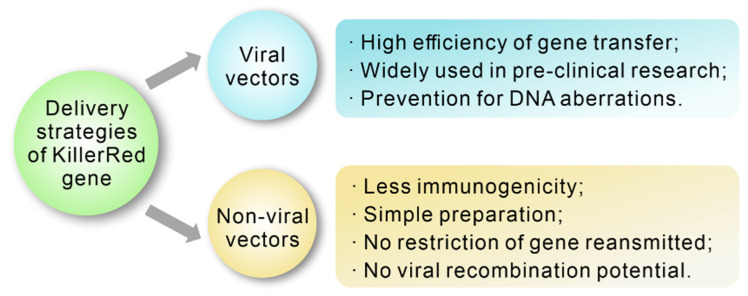
Advantages of diverse delivery strategies of KillerRed gene.

Nowadays, using chemical and physical methods to transfect specific genes in vitro has become more and more mature. However, an efficient and relative safe strategy of gene transfection in vivo is still a challenge. Since bare DNA cannot enter into cells due to its hydrophilic property, large size, and negative charge [60]. Recently, several promising approaches have been developed for transfecting the KillerRed gene into specific cells, including viral and non-viral delivery strategies (Figure 5).

#### 3.2.1. KillerRed Gene Delivery Based on Viral Vectors

Viral vectors with high transfection efficiency provide a promising method for temporary or permanent expressing genetic material into desired cells. Viral vectors are divided into integration vectors and non-integration vectors, depending on the presence/absence of the viral genome in the host cell [61]. Non-integrating vectors such as adeno-associated virus (AAV) vectors and adenovirus (Ad) vectors can avoid irreversible genomic-incorporation-caused DNA aberrations, and they have been widely used for gene transfection in pre-clinical experiments. Nowadays, researchers have proved that the delivery and infection of the KillerRed gene are precisely achieved in host species by non-integrated vectors.

Kiyoto Takehara et al. have designed a telomerase-specific recombinant adenovirus vector (TelomeKiller) to express KillerRed when it is activated by human telomerase reverse transcriptase (hTERT) promoter. Their studies have demonstrated that intratumor injection of TelomeKiller can inhibit the growth of non-small cell lung cancer and eliminate metastasis after the illumination of yellow-orange light (590 nm, 180 mW/cm^2^, 60 min) [62]. This recombinant vector is also efficient in eliminating human malignant melanoma after light stimulation (589 nm, 300 mW/cm^2^, 45 min) [63]. Moreover, a müller cells-specific adeno-associated virus vector expressed KillerRed in the vitreous of mice has been used to explore the changes in the structure and function of the retina after light stimulation (540–580 nm, 1000 lux, 60 min). The results shows that the activation of KillerRed leads to the loss of müller cells and then causes retinal degenerative disease. Meanwhile, it also suggests that KillerRed delivered by AAV vectors in müller cells may be useful to establish models of retinal dystrophies in large animals [64].

Viral vectors can deliver genetic materials into target cells due to their natural infectivity. However, the translation of specific genes is limited by insufficient capsid capacity, potential immunogenicity of the viral capsid, and insertional mutagenesis [65]. Thus, non-viral vectors for gene delivery have been attempted to overcome these roadblocks.

#### 3.2.2. KillerRed Gene Delivery Based on Non-Viral Vectors

Generally, non-viral vectors can be broadly defined as an assembly of cations that complex DNA into small-sized particles. Non-viral vectors have many advantages including simple preparation, low production cost, easy molecular structure manipulation, less immunogenicity, no restriction of genome material transmission, and no viral recombination potential [66], which is very efficient for gene delivery in vivo. Different categories of KillerRed gene delivery by non-viral vectors have been attempted.

**Figure 6:**
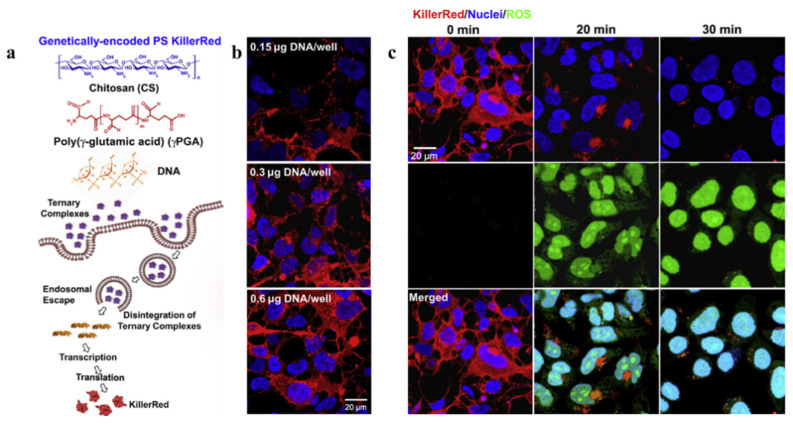
(**a**) Diagrams illustrating the functioning mechanism of a genetically encoded KillerRed complex. (**b**) KillerRed expression levels in HEK 293 cells treated with CS/DNA/g-PGA complexes containing differing concentrations of DNA. (**c**) Confocal images taken at the specified light exposure times demonstrating ROS output induced by photoactivation of KillerRed expressed in HEK293 cells. Reproduced with permission from Ref. [67]. Copyright 2013 Elsevier.

Cationic polymers such as chitosan and polyethylene are important carriers for negative genes delivery among varied non-viral gene vectors. They can interact with negatively charged KillerRed gene and further form positively charged particles. Chitosan (CS) can protect KillerRed gene from nuclease degradation, and poly (γ-glutamic acid) (γPGA) can enhance the expression of KillerRed by accelerating the intra-cellular unpackaging of CS/DNA complexes via electrostatic repulsion. Thus, the photosensitizing ternary complex consist of CS/pKillerRed/γPGA has been synthesized through an ionic-gelation method [65]. The study shows a decrease in both cell viability and membrane integrity of KillerRed-positive cells after irradiation (540–560 nm, 55 mW/cm^2^, 30 min) (Figure 6) [67]. Notably, the phototoxic reaction of KillerRed in cells gradually becomes negligible along with time, suggesting the biodegradability and safety of KillerRed. Except for chitosan, polyethylene (PEI) has also been used for KillerRed gene transfection. To enhance the cellular uptake of p53 and pKillerRed at acidic tumor microenvironment, researchers have designed the pH-responsive complex composed of plasmid DNAs, branched PEI and PEG-His-PEG-Glu. Evidence shows that a single administration dramatically decreased the development of tumors and increases the median animal lifespan from 28 days to 68 days with the illumination (593 nm, 100 mW/cm^2^, 20 min) [68].

However, cationic surface charge-mediated toxicity, incompatibility, and nonspecific interactions with blood components limit the application of cationic polymers [69]. Thus, cationic derivatives of natural polymers possess great application potentials for KillerRed gene delivery because of their low immunogenicity and toxicity. Pullulan is a well-known natural, neutral, and linear homopolysaccharide with availably chemical modification in hydroxyl groups. Jie Zhou et al. have synthesized cationic dendronized pullulan decorated with guanidine to improve KillerRed expression and ROS production to suppress cancer cell proliferation after photoactivation (532 nm, 4 W/cm^2^, 10 min) [69]. Moreover, the polysaccharide hydroxyethyl starch (HES) is also commonly used in hydrophilic, biocompatible, and biodegradable delivery systems [70,71]. CD-PGEA has the feature of excellent biocompatibility, non-immunogenicity, and low toxicity [72]. So, a genetic self-assembly nanosystem (HES@PGEA/pKillerRed-p53) has been designed to deliver pKillerRed-p53 and achieve the synergistic effect of p53 and KillerRed. Studies shows that the complex expressed better anti-tumor efficiency than monotherapy in 4T1 models after illumination (540–560 nm, 70 mW/cm^2^, 20 min) [73].

Although some progress has been made, further exploration of non-viral KillerRed gene delivery is still needed. As the transfection efficacy of non-viral vectors is often related to their toxicity [66], achieving safe and effective transfection is a key objective in the development of non-viral KillerRed gene delivery.

## 4. Applications of KillerRed Protein as an Exogenous Photosensitizer

**Figure 7:**
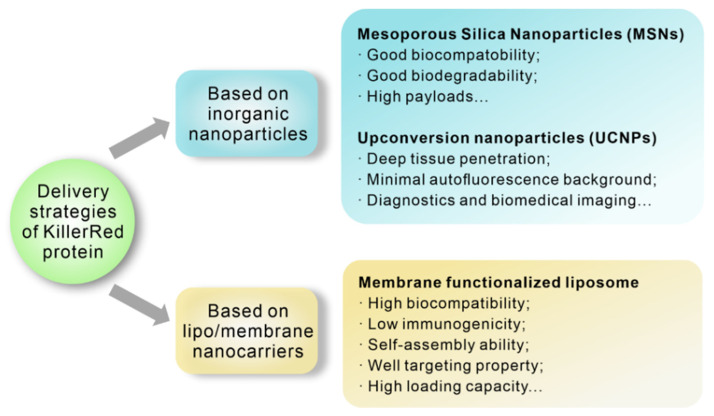
Advantages of diverse delivery strategies of KillerRed protein.

As mentioned previously, KillerRed can indeed serve as an endogenous photosensitizer and express at specific sites via gene delivery. However, gene therapy with endogenous KillerRed faces a problem of serious gene toxicity because of potential alteration of genetic composition. In contrast, using KillerRed protein as an exogenous photosensitizer can avoid such risks. For instance, studies have showed an effective inactivation of K562, NB4, and THP1 leukemia cells by purified KillerRed triggering cell apoptosis under light stimulation (400–780 nm, 80 mW/cm^2^, 20 min) [74]. Moreover, by injecting the *Escherichia coli* expressing KillerRed (KR-*E.coli*) into tumor tissue and illuminating with an appropriate wavelength (540–580 nm, 30 min), the CNE2 and HeLa tumors become necrotic and are eliminated without recurrence in two months [75]. However, there are still challenges for KillerRed protein delivery, such as instability during blood streaming or degradation by enzymes [76]. Various multifunctional nanoplatforms with tumor targets, deep tumor penetration, and effective cellular uptake have been developed to overcome these obstacles (Figure 7). In this section, the recent progress in the KillerRed protein delivery strategies will be separately described.

### 4.1. KillerRed Protein Delivery Based on Inorganic Nanoparticles

With technological development, nanoparticles have been designed using multiple agents from natural to synthetic materials [61]. Nanoparticles have many advantages such as high loading, good stability, and functional diversity modified with functional groups [77,78,79,80]. Therein, inorganic vehicles are using broadly in PDT to increase the selectivity and bioavailability of photosensitizers because of their high chemical stability and corrosion resistance under physiological conditions. The development of inorganic nanocarriers provides a new opportunity for KillerRed protein delivery.

#### 4.1.1. Mesoporous Silica Nanoparticles (MSNs)

**Figure 8:**
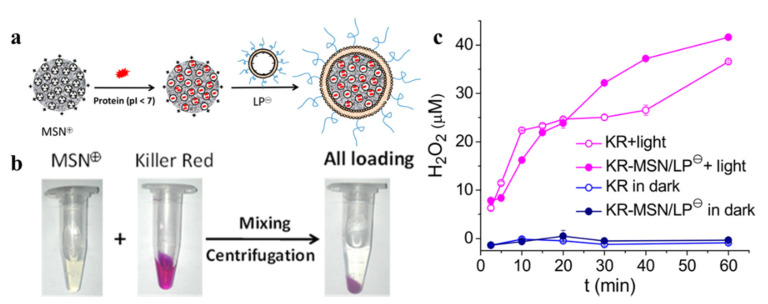
(**a**) Diagrams illustrating the synthesis of KR-MSN/LP. (**b**) The phenomenon of Killer Red protein-loaded MSN−. (**c**) H_2_O_2_ output from KR, KR-MSN/LP (KR content: 180 μM) after various periods of irradiation with LED light (10 mW/cm^2^). Reproduced with permission from Ref. [82]. Copyright 2019 American Chemical Society.

As promising nanocarriers for multiple therapies, MSNs offer many ideal drug delivery properties including biocompatibility, biodegradability, flexibility in size and shape, and porous structure for high payloads [81]. To achieve efficient and safe protein delivery, a multi-purpose selective system has been designed (Figure 8). The positively and negatively charged MSNs are prepared via an amine or carboxyl modification on their surface. Thus, several proteins including KillerRed can be loaded effectively into various MSNs through electrostatic interaction and pore absorption [82]. As an example, ROS can be detected by activating KR-MSN after LED illumination (10 mW/cm^2^, 60 min). This charge selective system achieves various proteins delivery in vitro. However, the short penetration depth of visible light limits the application of many photosensitizing proteins like KillerRed in vivo. Thus, more effective strategies still deserve to be explored.

#### 4.1.2. Upconversion Nanoparticles (UCNPs)

**Figure 9:**
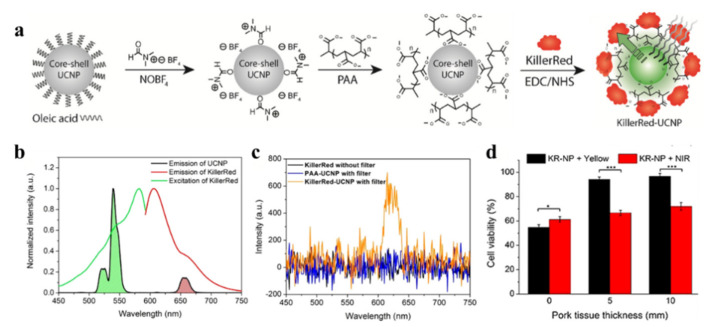
(**a**) Schematic of KillerRed-UCNP synthesis. (**b**) KillerRed standardized (green line) and spectra of emissions (red line) and UCNPs (black line) emission spectrum under 980 nm excitation. (**c**) KillerRed (black line), PAA-UCNP (blue line), and KillerRed UCNP (orange line) emission spectrum under 980 nm of arousal. (**d**) MDA-MB-231 cells treated with KillerRed-UCNP (200 μg/mL) irradiated for 30 min with various pork-tissue thicknesses mounted at the cell chamber, using a NIR laser (0.5 W/cm^2^) and yellow laser (0.2W/cm^2^). Reproduced with permission from Ref. [85]. Copyright 2017 Elsevier. *: *p* < 0.05; ***: *p* < 0.001.

The major obstacle of the application of KillerRed in biological tissues is the short excitation wavelength (~585 nm). The penetration depth of green light is typically less than 3 mm, which cannot achieve efficient tissue penetration [83]. The combination with upconversion nanoparticles (UCNPs) is one of the options to excite KillerRed with longer wavelengths. During the anti-stock emission process, UCNPs can convert low-energy light into high-energy light to achieve deep tissue penetration, minimal autofluorescence background, and diagnostics and biomedical imaging performance [84]. Nowadays, KillerRed has already been designed to covalently link with green-emitting UCNPs to enhance the therapeutic depth under NIR stimulation (Figure 9). NaYF_4_:Yb^3+^/Er^3+^ nanospheres emit visible luminescence at ~540 nm under NIR irradiation, and then the photon energy is transferred to KillerRed to generate ROS.

The results shows that after irradiation with NIR light (980 nm, 0.5 W/cm^2^, 30 min), the efficacy of PDT with KR-UCNP can reach about 70% at approximately 1cm tissue depth, while KillerRed only can just achieve about 7% [85]. The combination of KillerRed and UCNPs indeed presents new opportunities for the application of KillerRed in vivo. However, how to improve the loading efficiency of KillerRed, increase light-conversion efficiency, and reduce illumination time remain problems which limit the development of UCNP and photosensitizing proteins.

### 4.2. KillerRed Protein Delivery Based on Lipo/Membrane Nanocarriers

Liposomes, as common drug-encapsulating materials that consist of one or more bilayers of hydrophobic phospholipid around the core, present several distinctive characteristics including high biocompatibility, low immunogenicity, self-assembly ability, loading capacity for both hydrophilic and hydrophobic agents, and protection against cargo in physiological conditions [86]. Different liposomes have so far been widely studied as carriers of many agents, such as proteins or peptides, to improve cargo stability, extend systemic circulation and improve the accumulation of tumors [74]. Meanwhile, clinical applications of liposomes have been proved to be the most beneficial.

Liposomes have been applied widely for their unique advantages. Meanwhile, drug encapsulation in a liposome can ensure the regular use of drugs as the pharmacokinetic and pharmacodynamic properties can be controlled [86]. However, unexpected drug leakage from liposomes in vivo circulation may exert cytotoxic side effects and lead to failure of tumor eradication. The targeting moieties integrated into liposomes always require multiple chemical reactions and formulation processes that inevitably pose problems of low stability, poor reproducibility, and complicated assessments [87]. To overcome this limitation, Kim HY et al. have hybridized liposomes with the KillerRed-embedded cancer cell membrane (Lp-KR-CCM) where KillerRed cannot leak and ensure the cell source cancer is homotypic binding. In the case of homotypic tumor-bearing mice following green radiation (532 nm, 60 min), they embed LP-KR-CCM lipids adjuvants to promote an anticancer immune response [87]. The disadvantage of this approach lies in the weak penetration capacity of green light, which limits its application clinically and in deep tumors.

## 5. Comprehensive Therapy

**Figure 10:**
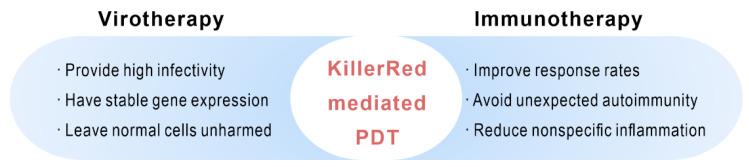
Advantages of KillerRed-mediated PDT combined with other therapies.

Tumor complexity, diversity, and heterogeneity severely limit the therapeutic potential of treatment. Multiply strategies such as chemotherapy, surgery, and immunotherapy have been widely used to treat a variety of neoplasms and have achieved excellent results. However, it cannot be completely ignored that the drawbacks of the single treatment such as the toxic side effects, tolerance of chemotherapeutic drugs, the incomplete surgical resection of surgery, and the weak efficacy of immunotherapy have limited the efficacy of cancer treatments [88]. As a non-invasive treatment approach, PDT has been applied to remove residual tissue in clinic because of its significant targeting and less damage to surrounding tissues. Photodynamic combinational therapies are continuously under development and provide new ideas for the diagnosis and treatment of cancer (Figure 10).

### 5.1. KillerRed-Mediated PDT Combined with Virotherapy

Virotherapy is an emerging approach by using biotechnology to convert viruses into therapeutic agents [89]. Compared to plasmid-based gene delivery, viral vectors can offer high infectivity and stable gene expression [90]. The U.S. Food and Drug Administration has approved clinical virotherapy use in cancer therapy [88]. However, most clinical trials of virotherapy are executed via intertumoral injection, which hinders the treatment of deep or metastatic tumors [91]. As a result, achieving efficacious and accurate systemic delivery is critical and will also promote the development of KillerRed in combination with virotherapy.

**Figure 11:**
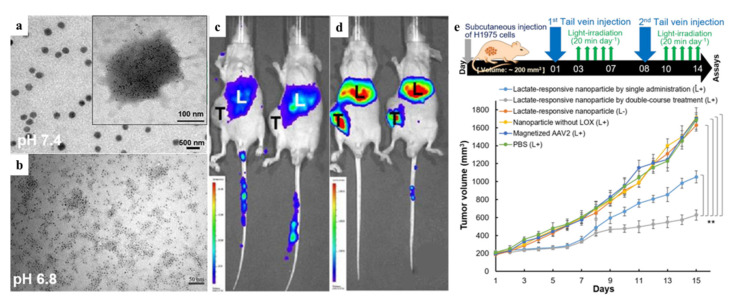
(**a**,**b**) Images from transmission electron microscopy (TEM) of lactate-responsive nanoparticles at various pH levels. (**c**,**d**) On the seventh day after tail vein injection of AAV2 alone (**c**) or lactate-responsive nanoparticles (**d**), IVIS photographs of mice were taken using AAV2- encoded luciferase as a detection signal. (**e**) Protocol for treating mice with H1975 xenografts (upper panel). Tumor volume (mm^3^) of different virus or nanoparticle-treated H1975 xenograft tumors through tail vein injection and/or tumor light irradiation (lower panel). Reproduced with permission from Ref. [94]. Copyright 2018 American Chemical Society. **: *p* < 0.05.

To achieve systemic delivery, several approaches have been attempted. At both the preclinical and clinical stages, recombinant adeno-associated virus serotype 2 (AAV2) has shown a good development prospect [92]. Furthermore, functionalized AAV2 nanoparticles can decrease the using dose, the risks of AAV-directed immune response, ectopic expression, and oncogene activation. Magnetic nanoparticles (MNPs) can accelerate vector accumulation at target sites and enhance virion infectivity via magnetic-field-enforced delivery [93]. As a result, a recombinant AAV2 carrying the KillerRed gene (AAV2-KR) chemically conjugated with iron oxide nanoparticles is designed to be guided in a magnetic field. The results show that PDT (laser, 1.5 mW/mm^2^, 20 min) with magnetic guidance significantly reduces tumor growth by inducing apoptosis [88]. This approach also proves that ironized AAV2-KR combined with PDT can successfully inhibit the growth of chemotherapy-resistant cancer cells [94]. Furthermore, a hypoxia-responsive carrier based on lactate production has been reported, which is self-assembled from hyaluronic acid (HA), 6-(2-nitroimidazole) hexylamine, lactate oxidase (LOX) and magnetized AAV2-KR. Within hypoxic and lactate-rich tumor microenvironments, LOX and magnetic fields can provide specific release. Meanwhile, LOX can catalyze lactate oxidation and produce H_2_O_2_ as end products that can induce bioreduction of HA and electrostatically dissociate the carrier to release AAV2-KR. Compared to control, the results show a significant limitation in tumor growth and a 2.44-fold reduction in tumor weight after a 2-week course after illumination (laser, 1.5 mW/mm^2^, 20 min) in vivo (Figure 11) [95].

There have been some attempts to combine PDT with virotherapy and improve therapeutic effectiveness. Actually, MNPs offer multiple opportunities for systemic delivery of viral vectors. However, as the low transfection efficiency remains an important factor limiting virotherapy development, further efforts are still demanded to develop efficient and safe gene delivery carriers.

### 5.2. KillerRed-Mediated PDT Combined with Immunotherapy

Some tumor cells can be elimination in conventional therapies and escape when they are resistance to the antitumor immune response. Immunotherapy, which is based on tumor escape mechanisms, manipulates the immune system to reactivate the antitumor immune response and overcome the pathways leading to escape [96]. Immunotherapy is now a powerful clinical strategy for cancer treatment and the number of new immunotherapy drugs approved each year is increasing with numerous treatments in clinical and preclinical development [97]. However, single immunotherapy still faces many challenges, including the low response rates to immunotherapy, unexpected autoimmunity, and nonspecific inflammation after the broad implementation of immunotherapy. It has been established that PDT can cause the release of antigen and immunogenic factors from dying tumor cells, such as damage-associated molecular patterns (DAMPs), which can promote DC maturation and activate an immune response against tumors. The distinct advantage of PDT makes it an appealing option compared with immunotherapy in cancer treatment [98]. Meanwhile, multifunctional nanoparticles have been proved efficient to improve immunotherapy potency, reduce toxic side effects, increase the accumulation within diseased tissues, and reduce off-target adverse effects. Thus, combining PDT with immunotherapy is of great significance for developing efficient and safe therapeutic approaches.

**Figure 12:**
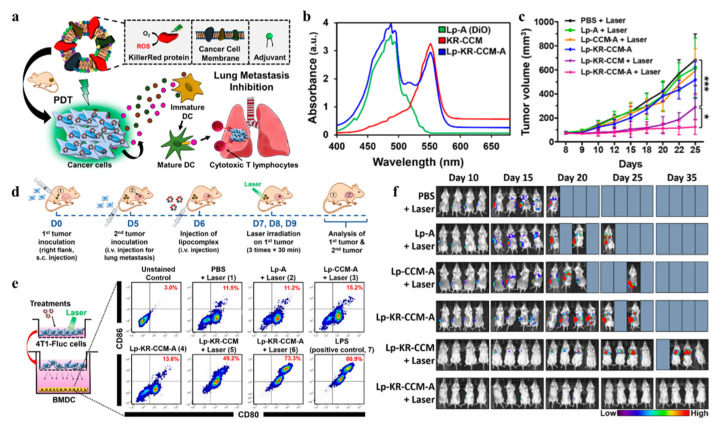
(**a**) Cancer treatment plan using lipocomplexes. (**b**) UV−vis spectra of the component of Lp-KR-CCM-A. (**c**) Primary tumor growth in mice. (**d**) Primary tumor inoculation to the right side, followed by Lp-KR-CCM-A-based PDT to ablate primary tumor and prevent lung metastasis. (**e**) In a co-culture method, multiple therapies and irradiation of 4T1-Fluc cells resulted in vitro BMDC maturation. (**f**) In vivo bioluminescence imaging was used to monitor the fate of inoculated luciferase-expressing 4T1-Fluc cancer cells in mice following multiple therapies for primary tumor ablation and lung metastasis prevention. Reproduced with permission from Ref. [87]. Copyright 2019 American Chemical Society. *: *p* < 0.05; ***: *p* < 0.001.

Previous studies have demonstrated that the expression of KillerRed can increase immunogenicity, providing a new idea for the combination of KillerRed-PDT and immunotherapy [99,100]. 4D5scFv-KillerRed has been designed with a specific anti-p185^HER−2-ECD^ antibody fragment 4D5scFv fused with KillerRed to retain both parts’ functional properties: high affinity to antigen and photodynamic activity. The results show that the recombinant protein has a good targeting property and can efficiently kill p185^HER−2-ECD^-expressing cancer cells when exposed to light (white light, 1 W/cm^2^, 10 min). Based on these results, further combination of cisplatin and the immunogenicity of KillerRed can activate the immune response and bring a remarkable additive effect to eliminate remaining malignant cells [101]. As mentioned previously, Kim HY et al. have hybridized liposomes with a cancer cell membrane embedded with KillerRed (Lp-KR-CCM). They then add monophosphoryl lipid A (MPLA), a lipid adjuvant, to stimulate an immune response by targeting TLR4 and elicit the maturation of dendritic cells (DCs). Maturated DCs are important in tumor killing because they secrete inflammatory cytokines and present tumor-associated antigens to T cells [102]. The primary tumor ablation and lung metastasis prevention have been observed after irradiation (532 nm, 60 min). Their results show potent antitumor activity and immune-activating both in vitro and in vivo experiments, indicating the promising prospect of PDT combined with immunotherapy (Figure 12) [87].

As an emerging photosensitive protein, KillerRed shows unique advantages and opportunities in combination therapies. However, the insufficient penetration depths and long-term irradiation increase the cost of KillerRed-PDT and also put forward higher requirements in clinical application. Developing promising functionalized nanoparticles with the advantages of “protection”, “responsiveness”, and “controlled release” may achieve better effects for immunotherapy.

## 6. Conclusions

**Figure 13:**
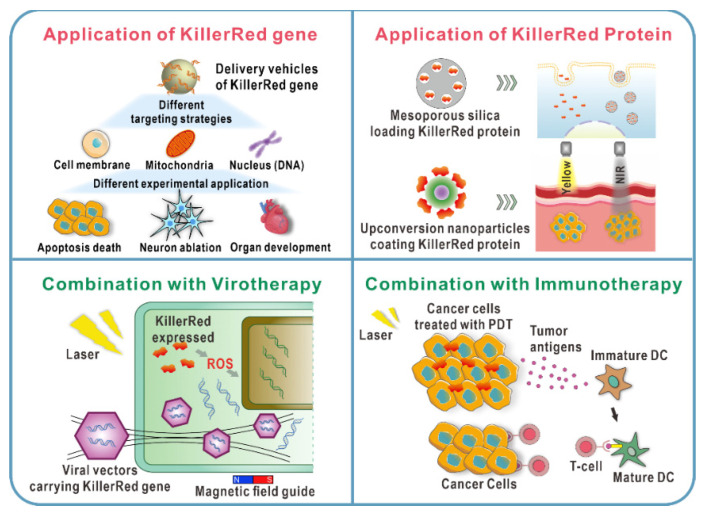
Schematic shows the application of KillerRed as both endogenous and exogenous photosensitizers. The combinations of PDT mediated by KillerRed and other therapies are showed.

PDT is a promising therapeutic option for many diseases, particularly cancer. However, it has not yet gained acceptance as a first-line treatment option due to the shortcomings of traditional PSs. KillerRed has shown more prominent advantages than traditional chemical photosensitizers and other applied photosensitizing proteins, which is mainly reflected in hydrophilicity, biocompatibility, better photostability, and higher ROS production. The application of KillerRed will be a promising approach for future technological breakthroughs in the field of PDT (Figure 13).

Higher expectations are always met with stricter requirements. To promote and accelerate the widely clinical applications of PDT in the future, there are still unresolved scientific issues and technical challenges for KillerRed, listed as follows:

(1) Systemic injection and local drug delivery are both important modes of administration. Of course, directly intertumoral injection of KillerRed vectors is also retained. Endogenous synthesis of KillerRed protein within the tumor can avoid side effects of off-target organs and maximize the efficiency of the therapy at the lesion location. Moreover, systemic administration offers unique advantages in the therapeutic process against tumor metastasis or deep tumor. Designing nanoscale drug delivery systems with tumor-targeting capability, controlled-release behavior, and responsiveness to the tumor microenvironment may be a good choice to efficiently utilize the biological function of KillerRed.
(2) Poor tissue penetration limits therapeutic efficacy and applicability of conventional PDT in the clinic. Combined with UCNP, KillerRed can be excited efficiently and achieve fluorescence imaging under NIR laser irradiation (expanding the light penetration depth to ≈1 cm). Meanwhile, the potential use of UCNP in UCL optical imaging, MRI, and CT achieve multimodal imaging guidance to provide precise structural information for unknown primary or metastatic tumor location, which finally achieve effective anticancer.
(3) Compared to monotherapy, photodynamic combination therapy, which is used for the majority of cancers, often yields better results. ROS generated by PDT can activate an acute inflammatory response, increase tumor immune prototype, promote drug delivery, and heighten local cytotoxicity to improve the efficacy of immunotherapy and chemotherapy. Mild photothermal therapy (mPTT) and sonodynamic therapy (SDT) will increase membrane permeability, enhance PSs uptake in tumor cells, and improve ROS aggregation to improve PDT efficiency. Meanwhile, combining with gene therapy can effectively delivery KillerRed into the specific site. PDT with other therapies, which has distinct benefits for primary cancers and peripheral metastatic tumors, can make a significant contribution to a systematic approach for cancer treatment.

## Figures and Tables

**Table 1:** Properties of the fluorescent proteins.

Category	Protein Photosensitizer	No. AA	Chromophore	λex [nm]	λem [nm]	Fluorescence Quantum Yield [φF]	^1^O_2_ Quantum Yield [φ^1^O_2_]	Photosensitized O_2_^−^ Formation
Fluorescent protein vatiant	KillerRed	239	QYG [13]	585	610	0.25 [12]	0.000 [14]	Y [15,16]
	KillerOrange	248	QWG [17]	512	555	0.42 [18]	-^1^	-
	SuperNova	271	QYG [19]	579	610	0.30 [19]	-	Y [19]
	TagRFP	237	MYG [20]	555	584	0.48 [21]	0.004 [22]	N [22]
Flavin-binding protein	miniSOG	106	-	448	528	0.37 [14]	0.03 [23,24]	Y [25]
	SOPP	106	-	440	487	0.43 [26]	0.25 [26]/0.39 [27]	Y [27]
	Pp2FbFPL30M	148	-	449	495	0.25 [28]	0.09 [29]	Y [28]

**Table 2:** Different targeting strategies of KillerRed in phototherapy.

Different Target Sites	Targeting Signal	Experimental Model	Parameters of Killerred Illumination			Ref.
			Wavelength	Optical Powers	Duration	
Membrane	Inserting membrane localization signal (MLS)	Zebrafish	535–575 nm	80 mW/cm^2^	2 h	[33]
		Zebrafish	Greenlight	-	0.5–1 h	[34]
		zebrafish	546–558 nm	100 W	20 min	[35]
		*Xenopus laevis*	545 nm	90 mW/cm^2^	18 h	[36]
		*Xenopus laevis*	545–565 nm	200 W	-	[37]
		*C. elegans*	426–593 nm	0.57–46 mW/cm^2^	0.1–2 h	[38]
		*C. elegans*	540–580 nm	269 mW/cm^2^	2 h	[39]
Mitochondria	Inserting mitochondria target sequence (MTS)	*C. elegans*	550–590 nm	100 mW/cm^2^	1 h	[40]
		*C. elegans*	543–593 nm	200–300 mW/cm^2^	1 h	[38]
		Rat and mouse hippocampal neuronal	Greenlight	120 W	1 h	[41]
		Mouse hippocampal neuronal and N2a neuroblastoma cells	561 nm	-	30 s	[42]
		HeLa cells	Visible light	-	1 h	[43]
		HEK293T and HeLa cells	530–610 nm	100 mW/cm^2^	20 min	[40]
		HeLa and SH-SY5Y cells	561 nm	-	-	[44]
Nuclear	Histone 2B	HeLa and Hela Kyoto cells	Greenlight	200 mW/cm^2^	15 min	[43]
	Histone 2A/Lamin B1	Hela and DU145 cells	Visible light	-	3 h	[45,46,47]
	Tet-repressor/Transcription activator	U2OS TRE and 263 cells	559 nm	150 mW/cm^2^	10 min	[48,49]
	Telomere-binding protein TRF1	U2OS, HeLa, MCF7, IMR90, and MCF7 cells	559 nm	15 W	0.33–4 h	[50,51]
	Telomere-binding protein TRF1/2	U2OS, 293, HeLa, and 293FT cells	Visible light	-	0.33–1 h	[35]

## References

[1] Wu H., Minamide T., Yano T. (2019). Role of photodynamic therapy in the treatment of esophageal cancer. Dig. Endosc..

[2] Morton C.A. (2018). A synthesis of the world’s guidelines on photodynamic therapy for non-melanoma skin cancer. G. Ital. Dermatol. Venereol..

[3] Railkar R., Agarwal P.K. (2018). Photodynamic Therapy in the Treatment of Bladder Cancer: Past Challenges and Current Innovations. Eur. Urol. Focus..

[4] Ikeda N., Usuda J., Kato H., Ishizumi T., Ichinose S., Otani K., Honda H., Furukawa K., Okunaka T., Tsutsui H. (2011). New aspects of photodynamic therapy for central type early stage lung cancer. Lasers. Surg. Med..

[5] Kaneko J., Kokudo T., Inagaki Y., Hasegawa K. (2018). Innovative treatment for hepatocellular carcinoma (HCC). Transl. Gastroenterol. Hepatol..

[6] Chilakamarthi U., Giribabu L. (2017). Photodynamic Therapy: Past, Present and Future. Chem. Rec..

[7] Ozog D.M., Rkein A.M., Fabi S.G., Gold M.H., Goldman M.P., Lowe N.J., Martin G.M., Munavalli G.S. (2016). Photodynamic Therapy: A Clinical Consensus Guide. Dermatol. Surg..

[8] Castano A.P., Demidova T.N., Hamblin M.R. (2005). Mechanisms in photodynamic therapy: Part two-cellular signaling, cell metabolism and modes of cell death. Photodiagnosis. Photodyn. Ther..

[9] Kwiatkowski S., Knap B., Przystupski D., Saczko J., Kędzierska E., Knap-Czop K., Kotlińska J., Michel O., Kotowski K., Kulbacka J. (2018). Photodynamic therapy—Mechanisms, photosensitizers and combinations. Biomed. Pharmacother..

[10] Abrahamse H., Hamblin M.R. (2016). New photosensitizers for photodynamic therapy. Biochem. J..

[11] Hilgers F., Bitzenhofer N.L., Ackermann Y., Burmeister A., Grünberger A., Jaeger K.E., Drepper T. (2019). Genetically Encoded Photosensitizers as Light-Triggered Antimicrobial Agents. Int. J. Mol. Sci..

[12] Bulina M.E., Chudakov D.M., Britanova O.V., Yanushevich Y.G., Staroverov D.B., Chepurnykh T.V., Merzlyak E.M., Shkrob M.A., Lukyanov S., Lukyanov K.A. (2006). A genetically encoded photosensitizer. Nat. Biotechnol..

[13] Carpentier P., Violot S., Blanchoin L., Bourgeois D. (2009). Structural basis for the phototoxicity of the fluorescent protein KillerRed. FEBS. Lett..

[14] Shu X., Lev-Ram V., Deerinck T.J., Qi Y., Ramko E.B., Davidson M.W., Jin Y., Ellisman M.H., Tsien R.Y. (2011). A genetically encoded tag for correlated light and electron microscopy of intact cells, tissues, and organisms. PLoS Biol..

[15] Pletnev S., Gurskaya N.G., Pletneva N.V., Lukyanov K.A., Chudakov D.M., Martynov V.I., Popov V.O., Kovalchuk M.V., Wlodawer A., Dauter Z. (2009). Structural basis for phototoxicity of the genetically encoded photosensitizer KillerRed. J. Biol. Chem..

[16] Vegh R.B., Solntsev K.M., Kuimova M.K., Cho S., Liang Y., Loo B.L., Tolbert L.M., Bommarius A.S. (2011). Reactive oxygen species in photochemistry of the red fluorescent protein “Killer Red”. Chem. Commun..

[17] Pletneva N.V., Pletnev V.Z., Sarkisyan K.S., Gorbachev D.A., Egorov E.S., Mishin A.S., Lukyanov K.A., Dauter Z., Pletnev S. (2015). Crystal Structure of Phototoxic Orange Fluorescent Proteins with a Tryptophan-Based Chromophore. PLoS ONE.

[18] Sarkisyan K.S., Zlobovskaya O.A., Gorbachev D.A., Bozhanova N.G., Sharonov G.V., Staroverov D.B., Egorov E.S., Ryabova A.V., Solntsev K.M., Mishin A.S. (2015). KillerOrange, a Genetically Encoded Photosensitizer Activated by Blue and Green Light. PLoS ONE.

[19] Takemoto K., Matsuda T., Sakai N., Fu D., Noda M., Uchiyama S., Kotera I., Arai Y., Horiuchi M., Fukui K. (2013). SuperNova, a monomeric photosensitizing fluorescent protein for chromophore-assisted light inactivation. Sci. Rep..

[20] Subach O.M., Malashkevich V.N., Zencheck W.D., Morozova K.S., Piatkevich K.D., Almo S.C., Verkhusha V.V. (2010). Structural characterization of acylimine-containing blue and red chromophores in mTagBFP and TagRFP fluorescent proteins. Chem. Biol..

[21] Merzlyak E.M., Goedhart J., Shcherbo D., Bulina M.E., Shcheglov A.S., Fradkov A.F., Gaintzeva A., Lukyanov K.A., Lukyanov S., Gadella T.W. (2007). Bright monomeric red fluorescent protein with an extended fluorescence lifetime. Nat. Methods..

[22] Ragàs X., Cooper L.P., White J.H., Nonell S., Flors C. (2011). Quantification of photosensitized singlet oxygen production by a fluorescent protein. Chemphyschem.

[23] Ruiz-González R., Cortajarena A.L., Mejias S.H., Agut M., Nonell S., Flors C. (2013). Singlet oxygen generation by the genetically encoded tag miniSOG. J. Am. Chem. Soc..

[24] Pimenta F.M., Jensen R.L., Breitenbach T., Etzerodt M., Ogilby P.R. (2013). Oxygen-dependent photochemistry and photophysics of “miniSOG,” a protein-encased flavin. Photochem. Photobiol..

[25] Barnett M.E., Baran T.M., Foster T.H., Wojtovich A.P. (2018). Quantification of light-induced miniSOG superoxide production using the selective marker, 2-hydroxyethidium. Free. Radic. Biol. Med..

[26] Westberg M., Holmegaard L., Pimenta F.M., Etzerodt M., Ogilby P.R. (2015). Rational design of an efficient, genetically encodable, protein-encased singlet oxygen photosensitizer. J. Am. Chem. Soc..

[27] Rodríguez-Pulido A., Cortajarena A.L., Torra J., Ruiz-González R., Nonell S., Flors C. (2016). Assessing the potential of photosensitizing flavoproteins as tags for correlative microscopy. Chem. Commun..

[28] Endres S., Wingen M., Torra J., Ruiz-González R., Polen T., Bosio G., Bitzenhofer N.L., Hilgers F., Gensch T., Nonell S. (2018). An optogenetic toolbox of LOV-based photosensitizers for light-driven killing of bacteria. Sci. Rep..

[29] Torra J., Burgos-Caminal A., Endres S., Wingen M., Drepper T., Gensch T., Ruiz-González R., Nonell S. (2015). Singlet oxygen photosensitisation by the fluorescent protein Pp2FbFP L30M, a novel derivative of Pseudomonas putida flavin-binding Pp2FbFP. Photochem. Photobiol. Sci..

[30] Vegh R.B., Bravaya K.B., Bloch D.A., Bommarius A.S., Tolbert L.M., Verkhovsky M., Krylov A.I., Solntsev K.M. (2014). Chromophore photoreduction in red fluorescent proteins is responsible for bleaching and phototoxicity. J. Phys. Chem. B.

[31] Shirmanova M., Yuzhakova D., Snopova L., Perelman G., Serebrovskaya E., Lukyanov K., Turchin I., Subochev P., Lukyanov S., Kamensky V. (2015). Towards PDT with Genetically Encoded Photosensitizer KillerRed: A Comparison of Continuous and Pulsed Laser Regimens in an Animal Tumor Model. PLoS ONE.

[32] Serebrovskaya E.O., Ryumina A.P., Boulina M.E., Shirmanova M.V., Zagaynova E.V., Bogdanova E.A., Lukyanov S.A., Lukyanov K.A. (2014). Phototoxic effects of lysosome-associated genetically encoded photosensitizer KillerRed. J. Biomed. Opt..

[33] Formella I., Svahn A.J., Radford R.A.W., Don E.K., Cole N.J., Hogan A., Lee A., Chung R.S., Morsch M. (2018). Real-time visualization of oxidative stress-mediated neurodegeneration of individual spinal motor neurons in vivo. Redox. Biol..

[34] The C., Korzh V. (2014). In vivo optogenetics for light-induced oxidative stress in transgenic zebrafish expressing the KillerRed photosensitizer protein. Methods. Mol. Biol..

[35] The C., Chudakov D.M., Poon K.L., Mamedov I.Z., Sek J.Y., Shidlovsky K., Lukyanov S., Korzh V. (2010). Optogenetic in vivo cell manipulation in KillerRed-expressing zebrafish transgenics. BMC Dev. Biol..

[36] Jewhurst K., McLaughlin K.A. (2019). Recovery of the Xenopus laevis heart from ROS-induced stress utilizes conserved pathways of cardiac regeneration. Dev. Growth. Differ..

[37] Jewhurst K., Levin M., McLaughlin K.A. (2014). Optogenetic Control of Apoptosis in Targeted Tissues of Xenopus laevis Embryos. J. Cell. Death..

[38] Williams D.C., Bejjani R.E., Ramirez P.M., Coakley S., Kim S.A., Lee H., Wen Q., Samuel A., Lu H., Hilliard M.A. (2013). Rapid and permanent neuronal inactivation in vivo via subcellular generation of reactive oxygen with the use of KillerRed. Cell. Rep..

[39] Kobayashi J., Shidara H., Morisawa Y., Kawakami M., Tanahashi Y., Hotta K., Oka K. (2013). A method for selective ablation of neurons in C. elegans using the phototoxic fluorescent protein, KillerRed. Neurosci. Lett..

[40] Shibuya T., Tsujimoto Y. (2012). Deleterious effects of mitochondrial ROS generated by KillerRed photodynamic action in human cell lines and C. elegans. J. Photochem. Photobiol. B.

[41] Ertürk A., Wang Y., Sheng M. (2014). Local pruning of dendrites and spines by caspase-3-dependent and proteasome-limited mechanisms. J. Neurosci..

[42] Grimm A., Cummins N., Götz J. (2018). Local Oxidative Damage in the Soma and Dendrites Quarantines Neuronal Mitochondria at the Site of Insult. Iscience.

[43] Petrova N.V., Luzhin A.V., Serebrovskaya E.O., Ryumina A.P., Velichko A.K., Razin S.V., Kantidze O.L. (2016). Inducing cellular senescence in vitro by using genetically encoded photosensitizers. Aging.

[44] Wang Y., Nartiss Y., Steipe B., McQuibban G.A., Kim P.K. (2012). ROS-induced mitochondrial depolarization initiates PARK2/PARKIN-dependent mitochondrial degradation by autophagy. Autophagy.

[45] Waldeck W., Mueller G., Wiessler M., Tóth K., Braun K. (2011). Positioning effects of KillerRed inside of cells correlate with DNA strand breaks after activation with visible light. Int. J. Med. Sci..

[46] Waldeck W., Mueller G., Glatting K.H., Hotz-Wagenblatt A., Diessl N., Chotewutmonti S., Langowski J., Semmler W., Wiessler M., Braun K. (2013). Spatial localization of genes determined by intranuclear DNA fragmentation with the fusion proteins lamin KRED and histone KRED und visible light. Int. J. Med. Sci..

[47] Lan L., Nakajima S., Wei L., Sun L., Hsieh C.L., Sobol R.W., Bruchez M., Van Houten B., Yasui A., Levine A.S. (2014). Novel method for site-specific induction of oxidative DNA damage reveals differences in recruitment of repair proteins to heterochromatin and euchromatin. Nucleic. Acids. Res..

[48] Whitefield D.B., Spagnol S.T., Armiger T.J., Lan L., Dahl K.N. (2018). Quantifying site-specific chromatin mechanics and DNA damage response. Sci. Rep..

[49] Nieborowska-Skorska M., Kopinski P.K., Ray R., Hoser G., Ngaba D., Flis S., Cramer K., Reddy M.M., Koptyra M., Penserga T. (2012). Rac2-MRC-cIII-generated ROS cause genomic instability in chronic myeloid leukemia stem cells and primitive progenitors. Blood.

[50] Tan R., Lan L. (2017). Induction of Site-Specific Oxidative Damage at Telomeres by Killerred-Fused Shelretin Proteins. Methods Mol. Biol..

[51] Sun L., Tan R., Xu J., LaFace J., Gao Y., Xiao Y., Attar M., Neumann C., Li G.M., Su B. (2015). Targeted DNA damage at individual telomeres disrupts their integrity and triggers cell death. Nucleic. Acids. Res..

[52] Zhang Y., Chen X., Gueydan C., Han J. (2018). Plasma membrane changes during programmed cell deaths. Cell. Res..

[53] Cheng H., Fan G.L., Fan J.H., Yuan P., Deng F.A., Qiu X.Z., Yu X.Y., Li S.Y. (2019). Epigenetics-inspired photosensitizer modification for plasma membrane-targeted photodynamic tumor therapy. Biomaterials.

[54] Jia H.R., Zhu Y.X., Xu K.F., Liu X., Wu F.G. (2018). Plasma membrane-anchorable photosensitizing nanomicelles for lipid raft-responsive and light-controllable intracellular drug delivery. J. Control. Release.

[55] Wang Z., Guo W., Kuang X., Hou S., Liu H. (2017). Nanopreparations for mitochondria targeting drug delivery system: Current strategies and future prospective. Asian. J. Pharm. Sci..

[56] Friedman J.R., Nunnari J. (2014). Mitochondrial form and function. Nature.

[57] Lin F., Bao Y.W., Wu F.G. (2018). Improving the Phototherapeutic Efficiencies of Molecular and Nanoscale Materials by Targeting Mitochondria. Molecules.

[58] Zielonka J., Joseph J., Sikora A., Hardy M., Ouari O., Vasquez-Vivar J., Cheng G., Lopez M., Kalyanaraman B. (2017). Mitochondria-Targeted Triphenylphosphonium-Based Compounds: Syntheses, Mechanisms of Action, and Therapeutic and Diagnostic Applications. Chem. Rev..

[59] Waldeck W., Mueller G., Wiessler M., Brom M., Tóth K., Braun K. (2009). Autofluorescent proteins as photosensitizer in eukaryontes. Int. J. Med. Sci..

[60] Oliveira A.V., da Costa A.M.R., Silva G.A. (2017). Non-viral strategies for ocular gene delivery. Mater. Sci. Eng. C Mater. Biol. Appl..

[61] Chen Y.H., Keiser M.S., Davidson B.L. (2018). Viral Vectors for Gene Transfer. Curr. Protoc. Mouse Biol..

[62] Takehara K., Tazawa H., Okada N., Hashimoto Y., Kikuchi S., Kuroda S., Kishimoto H., Shirakawa Y., Narii N., Mizuguchi H. (2016). Targeted Photodynamic Virotherapy Armed with a Genetically Encoded Photosensitizer. Mol. Cancer. Ther..

[63] Takehara K., Yano S., Tazawa H., Kishimoto H., Narii N., Mizuguchi H., Urata Y., Kagawa S., Fujiwara T., Hoffman R.M. (2017). Eradication of melanoma in vitro and in vivo via targeting with a Killer-Red-containing telomerase-dependent adenovirus. Cell. Cycle.

[64] Byrne L.C., Khalid F., Lee T., Zin E.A., Greenberg K.P., Visel M., Schaffer D.V., Flannery J.G. (2013). AAV-mediated, optogenetic ablation of Müller Glia leads to structural and functional changes in the mouse retina. PLoS ONE.

[65] Liao Z.X., Peng S.F., Chiu Y.L., Hsiao C.W., Liu H.Y., Lim W.H., Lu H.M., Sung H.W. (2014). Enhancement of efficiency of chitosan-based complexes for gene transfection with poly (γ-glutamic acid) by augmenting their cellular uptake and intracellular unpackage. J. Control. Release.

[66] Planul A., Dalkara D. (2017). Vectors and Gene Delivery to the Retina. Annu. Rev. Vis. Sci..

[67] Liao Z.X., Li Y.C., Lu H.M., Sung H.W. (2014). A genetically-encoded KillerRed protein as an intrinsically generated photosensitizer for photodynamic therapy. Biomaterials.

[68] Tseng S.J., Liao Z.X., Kao S.H., Zeng Y.F., Huang K.Y., Li H.J., Yang C.L., Deng Y.F., Huang C.F., Yang S.C. (2015). Highly specific in vivo gene delivery for p53-mediated apoptosis and genetic photodynamic therapies of tumour. Nat. Commun..

[69] Zhou J., Mohamed Wali A.R., Ma S., He Y., Yue D., Tang J.Z., Gu Z. (2018). Tailoring the Supramolecular Structure of Guanidinylated Pullulan toward Enhanced Genetic Photodynamic Therapy. Biomacromolecules.

[70] Yu C., Liu C., Wang S., Li Z., Hu H., Wan Y., Yang X. (2019). Hydroxyethyl Starch-Based Nanoparticles Featured with Redox-Sensitivity and Chemo-Photothermal Therapy for Synergized Tumor Eradication. Cancers.

[71] Yu C., Zhou Q., Xiao F., Li Y., Hu H., Wan Y., Li Z., Yang X. (2017). Enhancing Doxorubicin Delivery toward Tumor by Hydroxyethyl Starch-g-Polylactide Partner Nanocarriers. ACS. Appl. Mater. Interfaces.

[72] Ping Y., Hu Q., Tang G., Li J. (2013). FGFR-targeted gene delivery mediated by supramolecular assembly between β-cyclodextrin-crosslinked PEI and redox-sensitive PEG. Biomaterials.

[73] Xu C., Hu W., Zhang N., Qi Y., Nie J.J., Zhao N., Yu B., Xu F.J. (2020). Genetically multimodal therapy mediated by one polysaccharides-based supramolecular nanosystem. Biomaterials.

[74] Yuan M., Liu C., Li J., Ma W., Yu X., Zhang P., Ji Y. (2019). The effects of photodynamic therapy on leukemia cells mediated by KillerRed, a genetically encoded fluorescent protein photosensitizer. BMC Cancer.

[75] Yan L., Kanada M., Zhang J., Okazaki S., Terakawa S. (2015). Photodynamic Treatment of Tumor with Bacteria Expressing KillerRed. PLoS ONE.

[76] Liu X., Wu F., Ji Y., Yin L. (2019). Recent Advances in Anti-cancer Protein/Peptide Delivery. Bioconjug. Chem..

[77] Davis M.E., Chen Z.G., Shin D.M. (2008). Nanoparticle therapeutics: An emerging treatment modality for cancer. Nat. Rev. Drug. Discov..

[78] Master A., Livingston M., Sen Gupta A. (2013). Photodynamic nanomedicine in the treatment of solid tumors: Perspectives and challenges. J. Control. Release.

[79] Reddi E. (1997). Role of delivery vehicles for photosensitizers in the photodynamic therapy of tumours. J. Photochem. Photobiol. B.

[80] Bechet D., Couleaud P., Frochot C., Viriot M.L., Guillemin F., Barberi-Heyob M. (2008). Nanoparticles as vehicles for delivery of photodynamic therapy agents. Trends Biotechnol..

[81] Yu M., Gu Z., Ottewell T., Yu C. (2017). Silica-based nanoparticles for therapeutic protein delivery. J. Mater. Chem. B.

[82] Shi H., Liu S., Cheng J., Yuan S., Yang Y., Fang T., Cao K., Wei K., Zhang Q., Liu Y. (2019). Charge-Selective Delivery of Proteins Using Mesoporous Silica Nanoparticles Fused with Lipid Bilayers. ACS. Appl. Mater. Interfaces.

[83] Byrnes K.R., Waynant R.W., Ilev I.K., Wu X., Barna L., Smith K., Heckert R., Gerst H., Anders J.J. (2005). Light promotes regeneration and functional recovery and alters the immune response after spinal cord injury. Lasers. Surg. Med..

[84] Lucky S.S., Soo K.C., Zhang Y. (2015). Nanoparticles in photodynamic therapy. Chem. Rev..

[85] Liang L., Lu Y., Zhang R., Care A., Ortega T.A., Deyev S.M., Qian Y., Zvyagin A.V. (2017). Deep-penetrating photodynamic therapy with KillerRed mediated by upconversion nanoparticles. Acta. Biomater..

[86] Zununi Vahed S., Salehi R., Davaran S., Sharifi S. (2017). Liposome-based drug co-delivery systems in cancer cells. Mater. Sci. Eng. C Mater. Biol. Appl..

[87] Kim H.Y., Kang M., Choo Y.W., Go S.H., Kwon S.P., Song S.Y., Sohn H.S., Hong J., Kim B.S. (2019). Immunomodulatory Lipocomplex Functionalized with Photosensitizer-Embedded Cancer Cell Membrane Inhibits Tumor Growth and Metastasis. Nano Lett..

[88] Khalil A.S., Yu X., Xie A.W., Fontana G., Umhoefer J.M., Johnson H.J., Hookway T.A., McDevitt T.C., Murphy W.L. (2017). Functionalization of microparticles with mineral coatings enhances non-viral transfection of primary human cells. Sci. Rep..

[89] Leja J., Nilsson B., Yu D., Gustafson E., Akerström G., Oberg K., Giandomenico V., Essand M. (2010). Double-detargeted oncolytic adenovirus shows replication arrest in liver cells and retains neuroendocrine cell killing ability. PLoS ONE.

[90] Kim E., Oh J.S., Ahn I.S., Park K.I., Jang J.H. (2011). Magnetically enhanced adeno-associated viral vector delivery for human neural stem cell infection. Biomaterials.

[91] Miest T.S., Cattaneo R. (2014). New viruses for cancer therapy: Meeting clinical needs. Nat. Rev. Microbiol..

[92] Kotterman M.A., Schaffer D.V. (2014). Engineering adeno-associated viruses for clinical gene therapy. Nat. Rev. Genet..

[93] Liao Z.X., Kempson I.M., Fa Y.C., Liu M.C., Hsieh L.C., Huang K.Y., Wang L.F. (2017). Magnetically Guided Viral Transduction of Gene-Based Sensitization for Localized Photodynamic Therapy to Overcome Multidrug Resistance in Breast Cancer Cells. Bioconjug. Chem..

[94] Tseng S.J., Kempson I.M., Huang K.Y., Li H.J., Fa Y.C., Ho Y.C., Liao Z.X., Yang P.C. (2018). Targeting Tumor Microenvironment by Bioreduction-Activated Nanoparticles for Light-Triggered Virotherapy. ACS Nano..

[95] Tseng S.J., Huang K.Y., Kempson I.M., Kao S.H., Liu M.C., Yang S.C., Liao Z.X., Yang P.C. (2016). Remote Control of Light-Triggered Virotherapy. ACS Nano..

[96] Kennedy L.B., Salama A.K.S. (2020). A review of cancer immunotherapy toxicity. CA Cancer. J. Clin..

[97] Riley R.S., June C.H., Langer R., Mitchell M.J. (2019). Delivery technologies for cancer immunotherapy. Nat. Rev. Drug. Discov..

[98] Kleinovink J.W., van Driel P.B., Snoeks T.J., Prokopi N., Fransen M.F., Cruz L.J., Mezzanotte L., Chan A., Löwik C.W., Ossendorp F. (2016). Combination of Photodynamic Therapy and Specific Immunotherapy Efficiently Eradicates Established Tumors. Clin. Cancer. Res..

[99] Yuzhakova D.V., Shirmanova M.V., Serebrovskaya E.O., Lukyanov K.A., Druzhkova I.N., Shakhov B.E., Lukyanov S.A., Zagaynova E.V. (2015). CT26 murine colon carcinoma expressing the red fluorescent protein KillerRed as a highly immunogenic tumor model. J. Biomed. Opt..

[100] Serebrovskaya E.O., Yuzhakova D.V., Ryumina A.P., Druzhkova I.N., Sharonov G.V., Kotlobay A.A., Zagaynova E.V., Lukyanov S.A., Shirmanova M.V. (2016). Soluble OX40L favors tumor rejection in CT26 colon carcinoma model. Cytokine.

[101] Serebrovskaya E.O., Edelweiss E.F., Stremovskiy O.A., Lukyanov K.A., Chudakov D.M., Deyev S.M. (2009). Targeting cancer cells by using an antireceptor antibody-photosensitizer fusion protein. Proc. Natl. Acad. Sci. USA.

[102] Ma Y., Shurin G.V., Peiyuan Z., Shurin M.R. (2013). Dendritic cells in the cancer microenvironment. J. Cancer.

